# Predation stress experienced as immature mites extends their lifespan

**DOI:** 10.1007/s10522-022-09990-x

**Published:** 2022-09-09

**Authors:** Xiaoying Wei, Jianfeng Liu, Zhi-Qiang Zhang

**Affiliations:** 1 School of Biological Sciences, The University of Auckland, Auckland, New Zealand; 2 Guizhou Provincial Key Laboratory for Agricultural Pest Management of the Mountainous Region, Scientific Observing and Experimental Station of Crop Pest in Guiyang, Ministry of Agriculture, People’s Republic of China, Institute of Entomology, Guizhou University, Guiyang, 550025 People’s Republic of China; 3 Manaaki Whenua – Landcare Research, 231 Morrin Road, St Johns, Auckland, New Zealand

**Keywords:** Non-consumptive effects, Predation risk, Hormesis, Development, Fecundity, Lifespan

## Abstract

The early-life experience is important in modulating the late-life performance of individuals. It has been predicted that there were trade-offs between early-life fitness and late-life success. Most of the studies on senescence have focused on the trade-offs between the reproduction and lifespan, and the influences of diet, mating, and other factors. Because the negative, non-consumptive effects of predators could also modulate the behaviour and underlying mechanisms of the prey, this study aimed to examine the different effects of predator-induced stress experienced in the early life compared with later life of the prey. The prey (*Tyrophagus putrescentiae*) was exposed to predation stress from the predator (*Neoseiulus cucumeris*) during different periods of its life (immature, oviposition period, and post-oviposition period). The results showed that the predation stress experienced during immature stages delayed development by 7.3% and prolonged lifespan by 9.7%, while predation stress experienced in the adult stage (both oviposition and post-oviposition periods) decreased lifespans of *T. putrescentiae* (by 24.8% and 28.7%, respectively). Predation stress experienced during immature stages also reduced female fecundity by 7.3%, whereas that experienced during the oviposition period reduced fecundity of the prey by 50.7%. This study demonstrated for the first time lifespan extension by exposure to predation stress when young and highlighted the importance of early-life experience to aging and lifespan.

## Introduction

In predator–prey interactions, predators can affect prey both directly and indirectly, although indirect effects of predators, such as predation stress are not as well studied (e.g. Lemos et al. 2010; Dias et al. 2016). Predation stress has been shown to affect prey life history traits such as development (Peckarsky et al. 2002; Griffis-Kyle and Ritchie 2007; Thaler et al. 2012; Adler et al. 2013), and reproductive rates (Dahl and Peckarsky 2003; Zanette et al. 2011; Li and Zhang 2019a). Previous studies on predation risks focused mainly on the short-term effects on prey, such as development, behavior and reproduction (e.g., Warkentin 1995; Abrams and Rowe 1996; Oku et al. 2003; Choh et al. 2010; Rocha et al. 2020; Oliveira and Moraes 2021; Saavedra et al. 2022; Majchrzak et al. 2022). Recent studies on chronic predation stress have shown that predation stress could also reduce female lifespan and fecundity (Pan et al. 2018; Li and Zhang 2019a; Wei and Zhang 2022). In these studies on the effects of chronic predation stress, predator cues were exposed to the prey throughout their lifespan. We here test the hypothesis that the effects of predation stress might be reduced or even reversed if the level of stress is reduced by shorter duration of exposure (Wei and Zhang 2022). In this study, we reduced the duration of exposure of prey to predator cues to part of the lifespan of the prey and specifically compared the early- versus late-life effects of predation stress on prey fitness by applying predation stress during prey immature stages, oviposition period, and post-oviposition period.

As the resource available for organisms to use is always limited in any natural environment, they have to balance the allocation of gained energy to different life-history traits (Van Noordwijk and De Jong 1986; Houle 1991; Partridge and Barton 1993; Kokko 1998). For example, organisms need to balance the allocation of energy for fitness maintenance between early-life and later-life needs (Gurney and Nisbet 2004; Hughes and Reynolds 2005; Dmitriew 2011). This tradeoff is also one part of the disposable soma theory (Kirkwood and Holliday 1979; Kirkwood 1992). However, if there were adequate resource during the early life of individuals, it could provide benefits for individuals throughout their later life (Lindström 1999)—this is known as the “silver spoon” effects (Grafen and Clutton-Brock 1988). It has already been shown that sufficient resources in early life could be advantageous to a variety of life-history traits in the adulthood of individuals, such as survival rates, mating success, reproduction, and even immune function (e.g. Haywood and Perrins 1992; Birkhead et al. 1999; Tigreros 2013; Kelly et al. 2014; Kleinteich et al. 2015; Plesnar-Bielak et al. 2017; Griffin et al. 2018; Peters et al. 2019). In addition, if an individual invested more in early development, it could require less energy for body maintenance in the adulthood (Dmitriew 2011; Lee et al. 2011). Faster development generally indicates a decrease in lifespan and an increase in the rate of aging (Williams 1957; Monaghan et al. 2009). Many previous studies have focused on the effects of diet on the trade-offs between early reproduction and late senescence (e.g. Inness and Metcalfe 2008; Kwang et al. 2008; Blacher et al. 2017; Li and Zhang 2019b); and some studies have focused on the influences of diet on the trade-offs between early development and late lifespan (English and Uller 2016; Angell et al. 2020), and reproductive traits (Vega-Trejo et al. 2016). However, fewer studies have examined potential negative effects such as predation risks, which could also indirectly influence the trade-offs between early life and later life traits. Most previous studies on predation risk only focused on early-life traits of organisms. For instance, grasshoppers (*Ageneotettix deorum*) under the predation risk of spiders delayed development, achieved a smaller size at maturity and delayed the onset of reproduction (Danner and Joern 2004). The exposure to the predators, weasels (*Mustela nivalis*) and stoats (*M. erminea*), slowed the maturation rate of bank voles (*Clethrionomys glareolus*) and also delayed their reproduction (Heikkilä et al. 1993). Weaned females of wood mice (*Apodemus sylvaticus*) had lower growth rates and body mass under predation stress simulated by constant broadcasting of owl calls (Monarca et al. 2020). Moreover, there was also compensatory effects in the early life of predatory mites (*Phytoseiulus persimilis*) under transient intraguild predation (IGP) risk—the mites exposed to IGP risk during the larval stage delayed larval development but increased foraging activities and accelerated development during the next stage, which is the protonymph (Walzer et al. 2015).

Mite prey-predator systems are useful models for studying the effects of predation stress on prey life history traits including aging and lifespan (Li and Zhang 2019b). Behavioural responses of prey mites to predator cues have been well demonstrated: prey (*Tetranychus urticae*) increased activity and aggregation behaviour in response of cues of its predator *Phytoseiulus persimilis* (Hackl and Schausberger 2014; Dittmann and Schausberger 2017); prey mites (*T. urticae*) could avoid areas previously exposed to phytoseiid predators (Kriesch and Dicke 1997; Grostal and Dicke 1999); prey males (*Aculops allotrichus)* chose not to deposit spermatophores (Michalska 2016) and prey females (*T. urticae*) actively avoided depositing eggs in areas with signs of phytoseiid predators (Walzer and Schausberger 2012). Predation stress has also been shown to increase immature developmental time in prey (Freinschlag and Schausberger 2016; Li and Zhang 2019a; Wei and Zhang 2019, 2022), delay the onset of oviposition in prey (Hackl and Schausberger 2014), reduce the rates of oviposition in prey (Choh et al. 2010; Hackl and Schausberger 2014; Jacobsen et al. 2016; Li and Zhang 2019a; Wei and Zhang 2022), and shorten lifespan in prey (Li and Zhang 2019a; Wei and Zhang 2022).

**Fig. 1:**
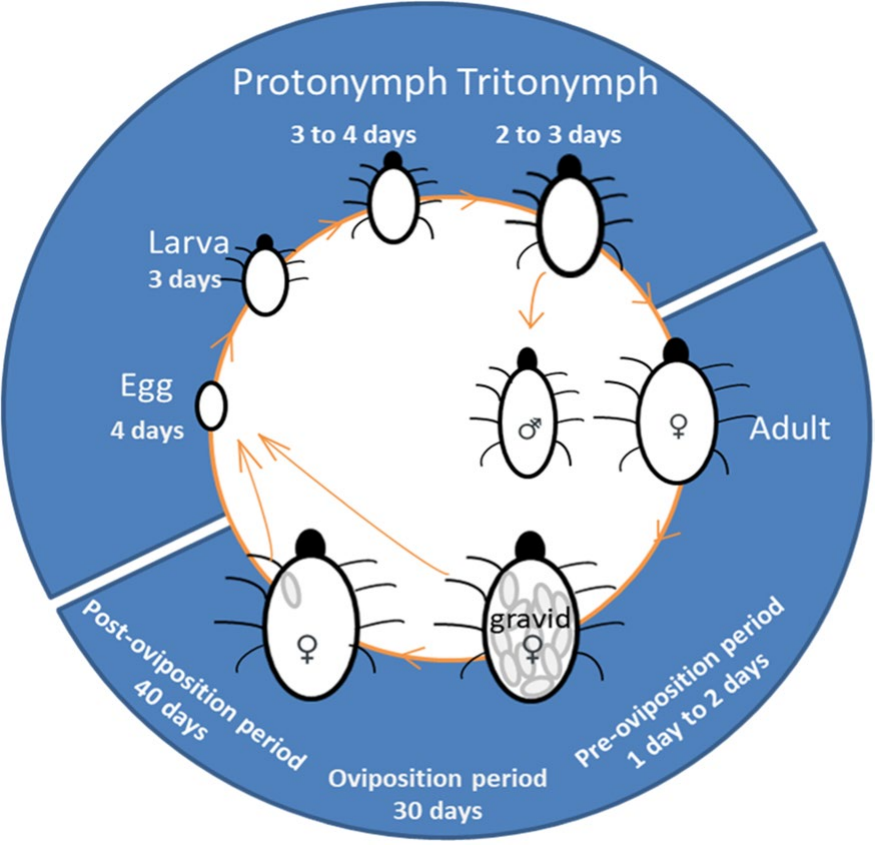
A typical life cycle of *Tyrophgus putrescentiae* at 25 ± 2 °C. Data are from control group in this experiment

In this study, we examined the effects of predation stress experienced during different life stages of the prey to demonstrate the differences in effects generated by exposure to predation stress in early versus in late life. The prey-predator system (*Tyrophagus putrescentiae* and *Neoseiulus cucumeris*) was used in this study because both species are easy to culture in the laboratory (Gu et al. 2020; Lee et al. 2020). *T. putrescentiae* is a common pest of stored food and young plants in greenhouses (Fan and Zhang 2007), while its predator *N. cucumeris* is a common biological control agent against various pest mites and small insects (Zhang 2003; McMurtry et al. 2013; Knapp et al. 2018). The immature phase of *T. putrescentiae* includes four stages (egg, larva, protonymph and tritonymph) and can be completed within a couple of weeks, whereas the adults live for two to three months, with a very short pre-oviposition period but long oviposition and post-oviposition periods (Fig. 1). A previous study using this system (Wei and Zhang 2019) demonstrated that it is relatively easy to apply constant predation stress. This model allows us to expose prey to predator-induced stress during immature development, the oviposition period or post-oviposition period, respectively, so that we can compare the influences on the life-history traits generated by predation risk experienced in early compared with late life.

## Materials and methods

### Mite colonies

The experimental colonies of the prey *T. putrescentiae* and its predator *Neoseiulus cucumeris* were derived from laboratory populations originally obtained from Bioforce Limited in South Auckland. The population of *T. putrescentiae* was kept in a transparent, cylindrical, plastic box (15 × 10 × 7 cm). They were fed dry yeast (*Saccharomyces cerevisiae* purchased from Goodman Fielder Limited, New Zealand). *Neoseiulus cucumeris* were mixed with bran plus *T. putrescentiae* in a transparent 1000-ml plastic box (15 × 10 × 7 cm) kept in a plastic container (35.5 × 23.5 × 12.0 cm) with water to prevent them from escaping. *Tyrophagus putrescentiae* were fed at about weekly intervals. Rearing containers of both the predator and prey species were placed in a cabinet with constant temperature (25 ± 2 °C), humidity (80 ± 2%), and photoperiod 16: 8 (L: D).

### Control group without predator-induced stress

Each individual mite was kept in a modified Munger cell. This was a rectangular plexiglass cell (25-mm wide, 38-mm long, and 3-mm thick) with a cone-shaped cell (top diameter 9 mm, bottom diameter 7 mm) in the middle. The bottom was covered with a mesh material (500 grids per square inch), using non-toxic glue (Think Creative, PVA Glue 500 g), which allowed the air exchange while preventing the escape of the mites. It was clipped together with a plexiglass of the same size without the cone-shaped cell on the top to keep the mite from escaping. A fine hair brush (size 000) was used to transfer a small droplet of the mixture of yeast and water into each Munger cell to feed the mites (details see Wei and Zhang 2019).

### Treatment group with predator-induced stress

**Fig. 2:**
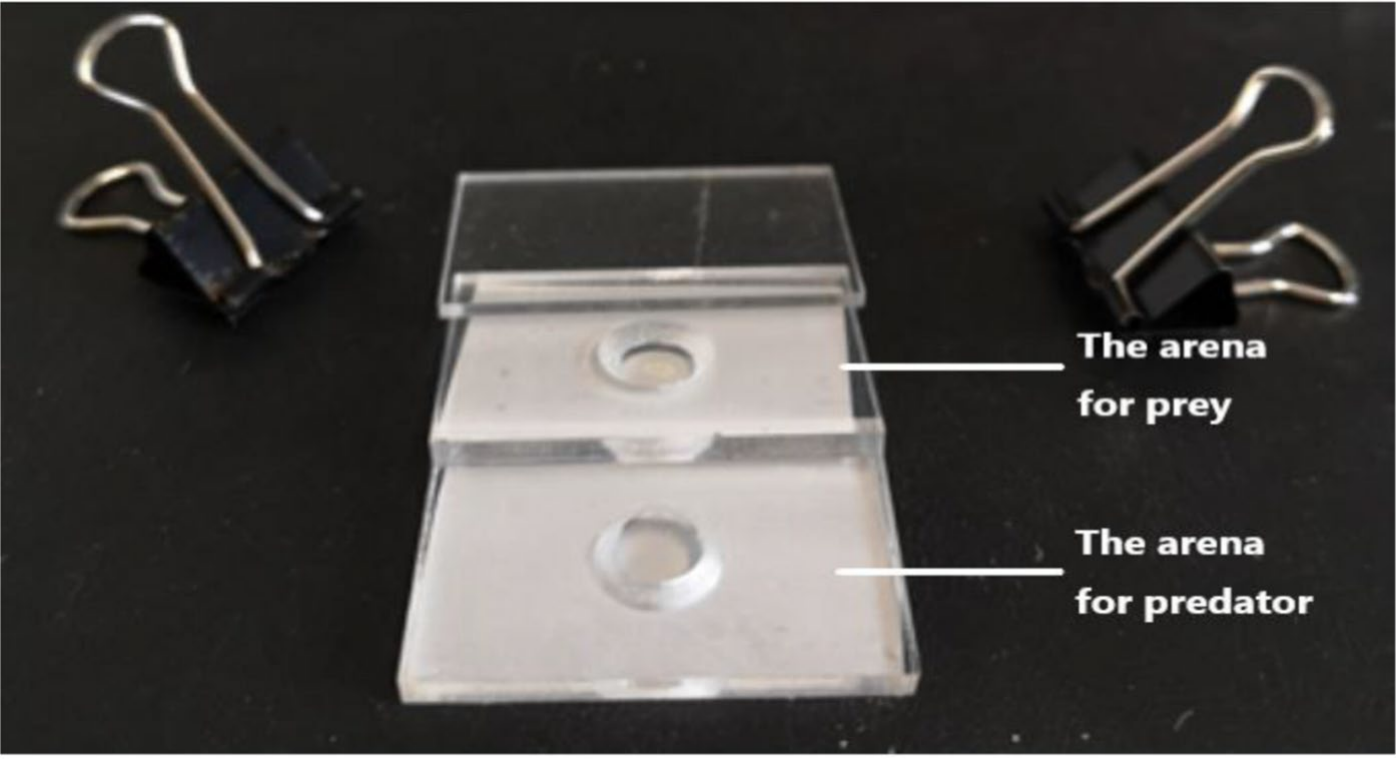
The experimental arenas for prey and predators in a modified Munger cell, showing partially stacked plexiglass slides with mesh cloth (white). The mesh fixed to the second slide separates the prey and predator arena, whereas the mesh fixed to the bottom slide allows ventilation. This figure was reproduced from Wei and Zhang (2019) with the permission of the publisher "Systematic & Applied Acarology Society"

The Munger cell (Fig. 2) of the treatment group consisted of three pieces of plexiglass of the same size as used in the control group. Two of pieces of plexiglass had a cone-shaped cell (top diameter 9 mm, bottom diameter 7 mm) in the middle; the third did not. The two pieces with the cell were clipped together, while their bottoms were pressed against each other and separated by a mesh material (500 grids per square inch). One plexiglass had a mesh material cover on the top to contain the predator mite, while the other without a cell was clipped on the top to contain experimental prey mite. The predatory mite and prey mite were therefore separated by the mesh material and could not contact each other; however, they could feel each other. The prey was fed a mixture of yeast and water, as was the control group. The predators were fed frozen eggs of the prey (details see Wei and Zhang 2019).

### Rearing units

In this study, each cell in the control group contained only a single prey, while all treatment groups had the prey and also five adult predators placed in a neighboring cell across a screen. The eggs of *T. putrescentiae* were picked up from the population and put into a larger cell (15 mm diameter) in a plexiglass slide (40 mm wide, 40 mm long, and 3 mm thick) applied with yeast, and similar age females were then collected from those matured eggs and kept for 24 h to lay eggs. These newly laid, 1-day-old eggs were collected and placed individually into each Munger cell. After they became adults, each prey was paired with its opposite sex of the same age and each cell contained only one couple. Their whole lifespan was observed and recorded. Prey food and the dead/inactive predators were replaced every 5–7 days.

There were four treatment groups, depending on when predator-induced stress was applied: (1) the immature stress group: the prey only had predation stress during their immature development; (2) the oviposition stress group: the prey in this group was given predation stress during their pre-oviposition and oviposition periods; (3) the postoviposition stress group: the prey was exposed to predation stress during the post-oviposition period; and (4) the full stress group: the prey was exposed to predation stress during their whole lifespan. Each group had 60 replicates at the beginning of the experiment. Records of life history parameters were made daily until all *T. putrescentiae* were dead. The dead individuals were then mounted in slides by using Hoyer’s medium, and dried in an oven heated at 50 °C for at least 2 weeks before measuring body size (represented by the length of the prodorsal plate) under a Nikon optical microscope (Li and Zhang 2018).

### Statistics

All data were analyzed by using R (version 4.1.1) and are available for open access from the DataStore of Landcare Research (10.7931/4kt2-c193). Although the data of the developmental times of the egg and larval periods conformed to homogeneity of variance, all other original data conformed neither to homogeneity, nor to normal distribution. Thus, the non-parametric Scheirer-Ray-Hare test, which is a two-factor analysis, was chosen to test the interactions between sex and treatment, and the influences of these two factors on the developmental time of the experimental mites. Similarly, as the lifespan data did not conform to normal distribution, the Scheirer-Ray-Hare test (Sokal and Rohlf 2012) was also used to analyze the prey’s lifespan. As the parameters of the oviposition period did not conform to homogeneity of variance and normal distribution, the non-parametric Kruskal–Wallis rank test (single-factor test) was used to test the effects of predation stress on the oviposition periods of the prey. Comparisons between any two groups were analyzed by Wilcoxon signed-rank test. The Cox proportional-hazards model was used for the survival analysis. Pairwise comparisons were analyzed by Kaplan–Meier survival analysis with log-rank test. Because the data of lifetime fecundity (number of eggs laid during the whole oviposition period), lifespan and body size were not normally distributed, The Scheirer-Ray-Hare test was also used to analyze the prey’s body size.

## Results

### Immature survival

Among the five groups (each started with 60 individuals), 58 individuals (32 females and 26 males) successfully developed to adults in the control group, 54 (28 females and 26 males) in the immature stress group, 53 (27 females and 26 males) in the oviposition stress group, 58 (37 females and 21 males) in the post-oviposition stress group, and 53 (29 females and 24 males) in the full stress group. Although different groups had different numbers of individuals developing to adults, the survival rates were all very high (88.3% to 96.7%) and were not statistically different among these groups (*χ*^2^ = 6.069, df = 4, *P* = 0.194). The egg hatching rates of different groups were the same as immature survival rates for these groups, because larvae and nymphs all developed to the next stage successfully.

### Development

**Fig. 3:**
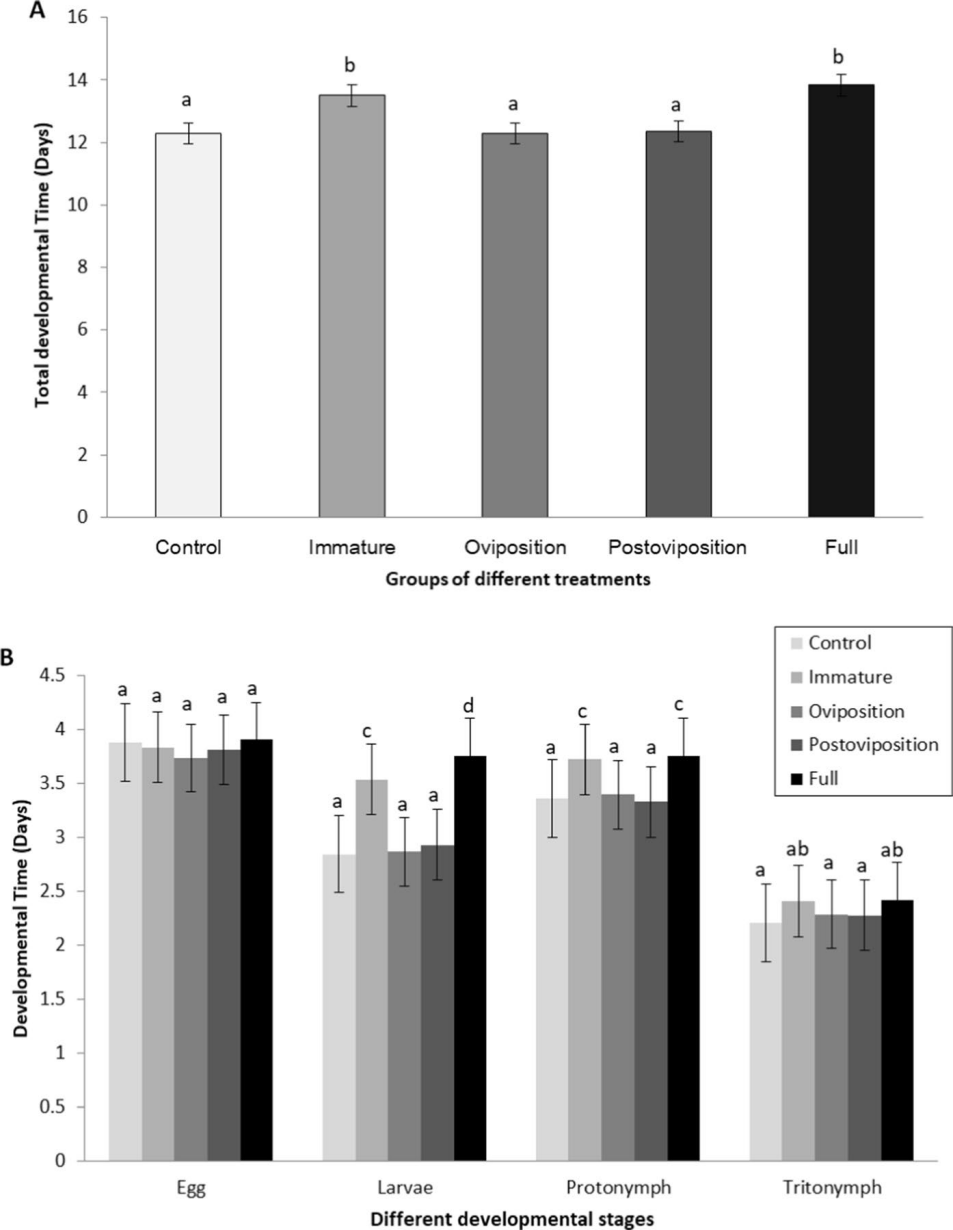
Developmental time of *T. putrescentiae* experienced predation stress during prey different stages. **A** Total developmental time (egg to adult) of *T. putrescentiae* experienced predation stress during different stages. **B** Developmental time of different developmental stages of *T. putrescentiae* under predation stress given during prey different stages. Bars with the same letters are not statistically different at *P* = 0.05. The error bars are standard errors

*Total development time* (Fig. 3A). There was no significant interaction between sex and treatment in their effects on developmental time (df = 4, *H* = 2.892, *P* = 0.576; Table 1). Overall, prey females took slightly longer time than males to develop to adults (df = 1, *H* = 81.384, *P* < 0.001), and individuals in the immature stress group and the full stress group took longer to become adults than other groups (df = 4, *H* = 94.156, *P* < 0.001). To be specific, the prey of the immature stress group took a similar time to the prey of the full group to reach maturity (*P* = 0.104), and both took a longer time than the prey of the control (*P* < 0.001).

**Table 1:** Total developmental time of *T. putrescentiae* females and males under predation stress of *Neoseiulus cucumeris* given during different prey stages

Total developmental time (days)	Control	Predation stress given during different stages				χ^2^	*df*	*P*
		Immature	Oviposition	Post-oviposition	Full			
Females	12.30 ± 0.67a	13.53 ± 1.07c	12.25 ± 1.05a	12.37 ± 0.85a	13.86 ± 1.05c	86.65	4	< 0.001
Males	12.27 ± 0.71a	13.49 ± 1.05c	12.27 ± 1.08a	12.35 ± 0.82a	13.80 ± 1.05c	50.61	4	< 0.001

Means (± se) in the same row with different letters are statistically different at *P* = 0.05

*Developmental durations for each life stage.* Egg incubation time was not different among treatments (all *P* > 0.05; Fig. 3B). The larvae and the protonymphs in immature stress group and in the full stress group developed slower than those in the control group (all *P* < 0.05). The tritonymph period in the immature stress group was similar with that in the full stress group (*P* > 0.05) but was longer than control and other groups (*P* < 0.05) (Fig. 3B).

### Reproduction

There were significant differences among treatment groups for different parameters of female reproduction, including total oviposition, daily oviposition, pre-oviposition period, oviposition period, and post-oviposition period (all *P* < 0.05, see Table 2).

**Table 2:** Reproductive parameters of *Tyrophagus putrescentiae* under predation stress of *Neoseiulus cucumeris* given during different prey stages

Parameters	Control	Predation stress given during different stages:				*P*
		Immature	Oviposition	Post-oviposition	Full	
Pre-oviposition period (days)	1.31 ± 0.09a	1.38 ± 0.10a	1.68 ± 0.11a	1.33 ± 0.11a	1.67 ± 0.14a	*P* = 0.039
Oviposition period (days)	29.42 ± 0.33a	27.65 ± 0.23b	28.48 ± 0.31a	29.38 ± 0.43a	26.42 ± 0.21c	*P* < 0.001
Post-oviposition period (days)	38.31 ± 2.10a	42.96 ± 1.58a	19.6 ± 1.45b	13.71 ± 1.9c	10.75 ± 1.57c	*P* < 0.001
Daily reproductive rate (eggs/female/day)	14.41 ± 0.14a	14.22 ± 0.12a	7.35 ± 0.12b	14.29 ± 0.23a	7.76 ± 0.1c	*P* < 0.001
Fecundity (eggs/female)	423.46 ± 4.52a	392.73 ± 2.69b	208.72 ± 2.64c	418.71 ± 5.45a	204.83 ± 2.73c	*P* < 0.001

Means (± se) in the same row with same letters are not statistically different at *P* = 0.05

Specifically, the fecundity in the oviposition stress group was similar to that in the full stress group (*P* = 0.437). The females of the immature stress group, however, laid significantly fewer eggs than the females in the control group (*P* < 0.001), but more eggs than the females in the oviposition stress group (*P* < 0.001) and the full stress group (*P* < 0.001). Thus, predation stress experienced during the immature stage reduced fecundity but not as much as that experienced during oviposition period.

The females in the immature stress group produced as many as eggs per day as those in the control group (*P* = 0.996). Additionally, the oviposition stress group had a slightly lower reproductive rate than the full stress group (*P* = 0.016), while both of these two groups had lower daily oviposition (nearly a half) than the other three groups (all *P* < 0.001). Thus, only predation stress experienced during oviposition period reduced daily reproductive rates.

The oviposition period of the females in the control was similar to those in the oviposition stress group (*P* = 0.173). The females in the immature stress group, however, had a shorter oviposition period than those in the control group (*P* = 0.001), and the oviposition stress group (*P* = 0.026), while they had longer oviposition periods than the females of the full stress group (*P* = 0.002). Thus, predation stress experienced during the immature stage reduced the duration of the oviposition period.

The females in the immature stress group did not live longer after laying eggs than those in the control group (*P* = 0.167). Additionally, the post-oviposition periods in the postoviposition stress group and the full stress group had approximately similar durations (*P* = 0.231). Moreover, the post-oviposition period in the oviposition stress group was longer than the full stress group (*P* < 0.001) and the post-oviposition group (*P* = 0.049), while it was shorter than those of the control group (*P* < 0.001) and the immature stress group (*P* < 0.001). Thus, predation stress experienced during the post-oviposition period reduced the duration of the post-oviposition more than that experienced during oviposition period.

Although the treatment effect on the pre-oviposition period was just statistically significant (*P* = 0.039), the non-parametric pairwise comparisons were not powerful enough to show different between the treatments (all *P* > 0.05; Table 2).

### Survival and lifespan

**Fig. 4:**
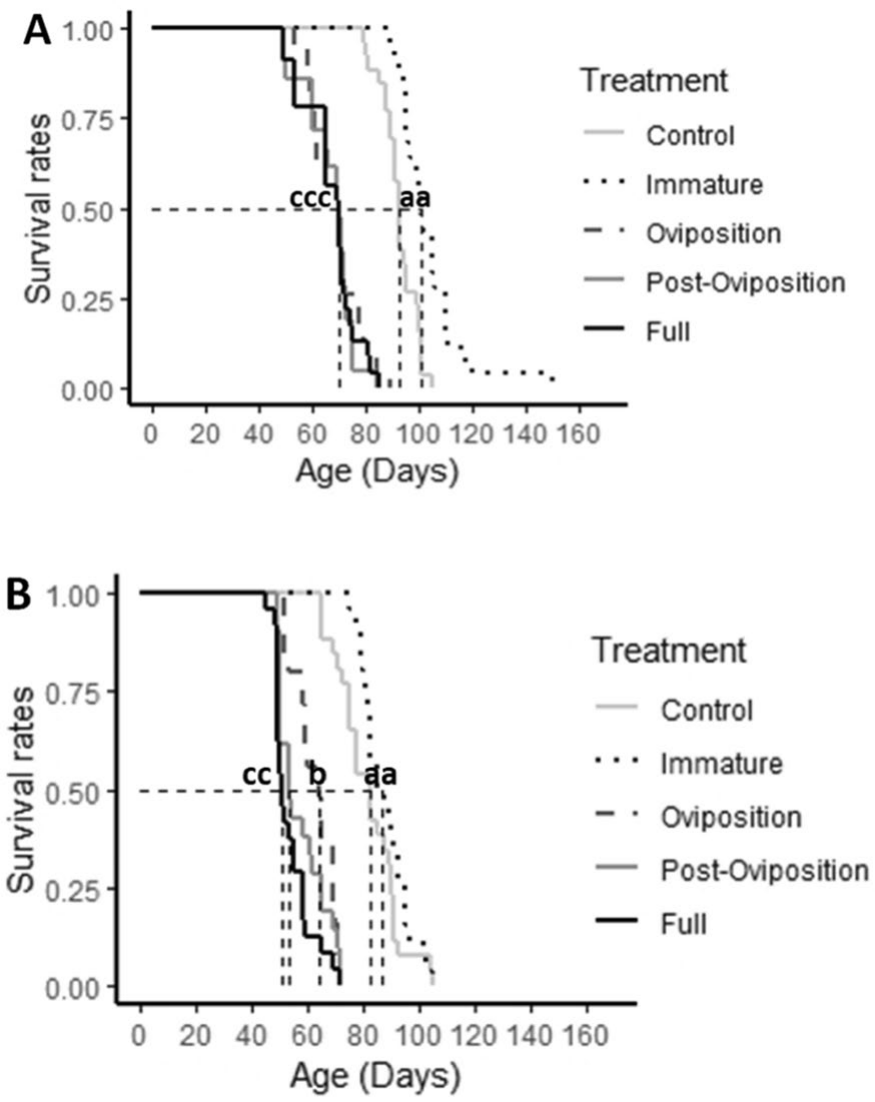
**A** Survival rates of males of *T. putrescentiae* experienced predation stress during different prey stages. **B** Survival rates of females of *T. putrescentiae* subjected to predation stress during different prey stages. survival curves with the same letters are not statistically different at *P* = 0.05

The survival rates of individuals in the oviposition stress group, the post-oviposition stress group, and the full stress group declined faster than those in the control group and the immature group in both males (χ^2^ = 129, df = 4, *P* < 0.001, Fig. 4A) and females (*χ*^2^ = 136, df = 4, *P* < 0.001, Fig. 4B). However, the males and the females of the immature stress group and the oviposition stress group behaved differently. To be specific, the males in the oviposition stress group had similar survival rates to those in the post-oviposition stress group (*χ*^2^ = 0.5, df = 1, *P* = 0.5) and the full stress group (*χ*^2^ = 0.2, df = 1, *P* = 0.6). However, although the females in the oviposition stress group had similar survival rates to the females in the post-oviposition stress group (*χ*^2^ = 2.3, df = 1, *P* = 0.1), their survival rate was higher than the females in the full stress group (*χ*^2^ = 12.6, df = 1, *P* < 0.001). Moreover, the males in the immature stress group had greater survival rates than the males in the control group (*χ*^2^ = 18, df = 1, *P* < 0.001), whereas the females in the immature stress group had similar survival rates to the females in the control group (*χ*^2^ = 2.1, df = 1, *P* = 0.1).

**Fig. 5:**
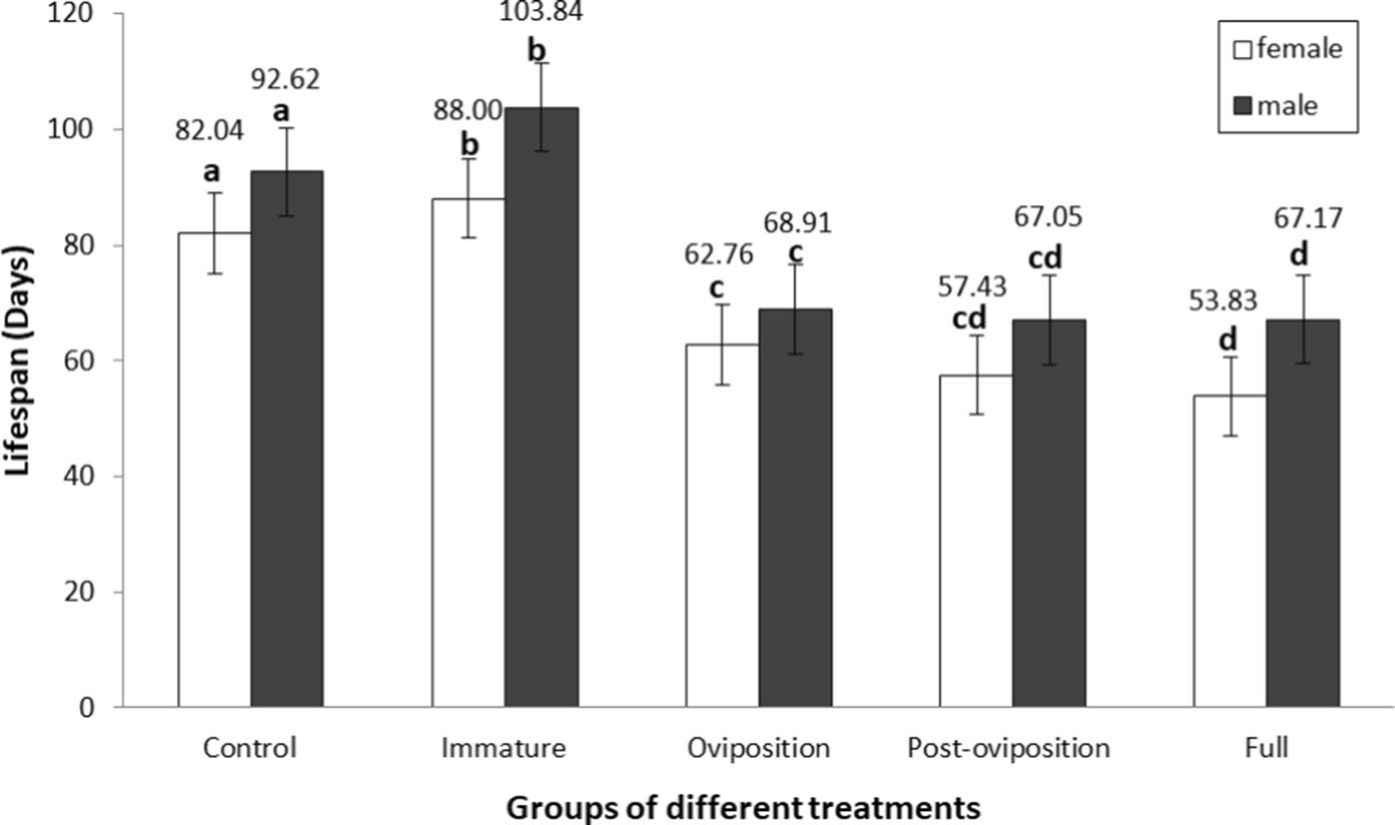
Lifespan of males and females of *T. putrescentiae* that experienced predation stress during different prey stages. Bars in the same colour with the same letters are not statistically different at *P* = 0.05

Although the males in each group lived longer than the females (*W* = 4652, *P* < 0.001; Fig. 5), the interaction between sex and treatment was not significant (df = 4, *H* = 1.183, *P* = 0.88). There was no difference in lifespan between the oviposition stress group and the post-oviposition stress group (*W* = 1174, *P* = 0.179). While similar trends had been found between the post-oviposition stress group and the full stress group (*W* = 849.5, *P* = 0.259), individuals of the oviposition stress group lived longer than the individuals of the full stress group (*W* = 779, *P* = 0.009). Additionally, the individuals in the immature stress group lived longer than the individuals in the control group (*W* = 823.5, *P* < 0.001), whereas the individuals of the oviposition stress group lived shorter than those of the control group (*W* = 2330.5, *P* < 0.001).

### Body size

The gender effects on body size were highly significant (*P* < 0.001), showing larger females than males (Table 3). There were no effects of treatments on body size (*P* = 0.999;). There was also no interaction between treatments and gender (*P* = 0.996).

**Table 3:** Body size (prodorsal shield length) of *Tyrophagus putrescentiae* under predatory stress of *Neoseiulus cucumeris* given during different prey stages

Body length (µm)	Control	Predation stress given during different stages:				χ^2^	*df*	*P*
		Immature	Oviposition	Post-oviposition	Full			
Females	88.64 ± 4.41	89.25 ± 6.34	88.52 ± 4.48	88.57 ± 4.46	87.98 ± 3.76	0.439	4	0.979
Males	73.21 ± 3.18	73.04 ± 3.19	73.45 ± 2.56	73.14 ± 2.81	73.63 ± 1.90	0.458	4	0.977

Data in the format of means ± se

## Discussion

Predator-induced stress has already been shown to affect a variety of prey life-history traits (e.g. Peckarsky et al. 2002; Dahl and Peckarsky 2003; Griffis-Kyle and Ritchie 2007; Zanette et al. 2011; Thaler et al. 2012; Adler et al. 2013; Li and Zhang 2019a). It is also well known that there can be trade-offs in resource allocation between early-life and late-life traits when the resource is often limited (e.g. Gurney and Nisbet 2004; Hughes and Reynolds 2005; Dmitriew 2011). Many previous studies have focused either on the trade-offs between early reproduction and late lifespan (e.g. Robinson et al. 2006; Lemaître et al. 2014; Travers et al. 2015; Adler et al. 2016; Rodríguez-Muñoz et al. 2019), or on the influences on the trade-offs generated by factors such as diets (e.g. English and Uller 2016; Angell et al. 2020). To our knowledge, this study is the first to involve the periodic predation pressure applied during different stages of prey life history to examine the relationship between early development, reproduction, and late senescence. One of the most important findings of this study is that the early-life immature experience of predation risk could show totally opposite results in lifespan compared with the late-life effects of predation risk.

It has been shown that females and males seem to involve different strategies in development: females were likely to mature at bigger size to ensure enough energy for the following reproduction, while males tend to develop fast to defend against predators and compete with peers to gain more chances of mating (De Block and Stoks 2003; Mikolajewski et al. 2005). In agreement with this, we found that *T. putrescentiae* showed obvious sex-specific responses in body size and developmental time: females generally matured slower at bigger sizes, whereas males developed faster at a smaller size. Although many previous studies suggested prey under predation risk developed fast and had a relatively lower growth rate (McPeek et al. 2001; Altwegg 2002; Dahl and Peckarsky 2003; Griffis-Kyle and Ritchie 2007; Thaler et al. 2012; Clinchy et al. 2013), our study showed no significant difference in body size between the control group and stress groups. However, the males and females had delayed development under predation risk, which is consistent with results in some other species (Beketov and Liess 2007; Li and Zhang 2019a).

Most studies on aging suggested the tradeoff ‘live fast, die young’ (Hunt et al. 2004; Monaghan et al. 2009; Bestion et al. 2015; Travers et al. 2015; Hooper et al. 2017). Our results showed that the predation stress given in earlier life during immature development could actually delay development, extend lifespan, and increase survival rates in later life, whereas the pressure given during oviposition and post-oviposition periods in later life could shorten lifespan and decrease survival rate. Individuals in the immature group increased their lifespans by 9.7% compared with the individuals in the control, whereas the individuals in oviposition group, post-oviposition group, and full group decreased their lifespans by 24.8%, 28.7%, and 30.9%, respectively. The results of predation pressure supplied in later life and during the whole lifespan were consistent with that of a previous study on spider mites that also showed that the females had an increase in development time and a reduction in lifespan under predation stress applied throughout the lifespan (Li and Zhang 2019a). Reduced diet quality in flies in earlier life could also result in delayed development; however, the fast-developed males under a high quality diet had longer lifespans but aged faster (Angell et al. 2020). Our results of early-life experience of predation stress were similar to the effects of heat or cold shock on life history in literature on hormesis: e.g. early-life heat shock could slow development and prolong nematode lifespan (Lind et al. 2017) and increase longevity and heat resistance in male *Drosophila* (Hercus et al. 2003), whereas early-life cold shock extended lifespan of male fruit flies (Le Bourg 2007). A further study on *Drosophila* showed that a mild stress at early as well as older ages could increase fly resistance to strong stresses with positive effects on longevity (Le Bourg 2011). All these studies suggest that the trade-offs between development and aging are complex, especially for predation risk because it can influence the prey by involving a variety of underlying mechanisms, such as the neurochemistry (Nanda et al. 2008), gene expression (Zhang et al. 2009), amygdala (Choi and Kim 2010), and glucocorticoid levels (Creel et al. 2009; Thaler et al. 2012). This study, however, showed that the results of predation stress in the early life and later life were totally different: although early-life predation stress delayed the development of the prey, it also generated benefits in increasing survival rates in later life and extending lifespan.

Moreover, compared with the control group, the individuals of the immature stress group had a slight reduction in fecundity—reducing by only 7.26%—while the individuals of the oviposition stress group and full group had reduced fecundity dramatically by 50.7% and 51.8%. However, contrary to the previous studies, which suggested there were trade-offs between reproduction and lifespan (Adler et al. 2013, 2016; Rodríguez-Muñoz et al. 2019), our results showed no correlation between fecundity and lifespan of the prey. It only showed that the predation stress has not only reduced the fecundity, but also decreased lifespan, which confirms the results of a previous study that female mites under predation stress had a reduction in fecundity and lifespan (Li and Zhang 2019a). This may demonstrate that the mites failed to balance the energy use under the relatively long-term predation risk (Li and Zhang 2019a). In addition, the males in the oviposition group had a similar trend in survival rates to males in the post-oviposition group and in the full group, whereas females in the oviposition stress group had reduced survival rates, although the reduction of their survival rates was less than the females in the full stress group and post-oviposition group. This may indicate that gravid females were more sensitive during the oviposition period, and the dramatic reduction of fecundity might release the pressure and result in a lower reduction of survival rates.

To conclude, it is obvious from this study that the predation pressure experienced during the immature stage could extend the lifespans of the prey while the predation pressure received during the adult stage (both oviposition and post-oviposition periods) decreased the lifespans of the prey. As the later periods were at least twice as long as the former period, the relatively long-term chronic predation stress may be still too strong for the prey. It will be rewarding to explore the effects on lifespan of even shorter-term exposure to predation stress (i.e. a matter of a few days) in future studies. It is also interesting and necessary to carry on studies at molecular level. For example, future studies could also compare proteomes or transcriptomes between controls and immature groups after immature development. This will provide us a better understanding of the molecular mechanisms of aging influenced by predation stress.
